# The ecto-nucleotide pyrophosphatase/phosphodiesterase 2 promotes early osteoblast differentiation and mineralization in stromal stem cells 

**DOI:** 10.1152/ajpcell.00692.2023

**Published:** 2024-01-15

**Authors:** Irina L. Tourkova, Quitterie C. Larrouture, Silvia Liu, Jianhua Luo, Paul H. Schlesinger, Harry C. Blair

**Affiliations:** ^1^Research Service, VA Medical Centre, Pittsburgh, Pennsylvania, United States; ^2^Department of Pathology, University of Pittsburgh, Pittsburgh, Pennsylvania, United States; ^3^Department of Cell Biology, Washington University, St. Louis, Missouri, United States

**Keywords:** bone mineralization, ecto-nucleotide pyrophosphatase/phosphodiesterase 2, osteoblast, osteoblast differentiation, stromal stem cell

## Abstract

The phosphodiesterase enzymes mediate calcium-phosphate deposition in various tissues, although which enzymes are active in bone mineralization is unclear. Using gene array analysis, we found that a member of ecto-nucleotide pyrophosphatase/phosphodiesterase family, ENPP2, was strongly down-regulated with age in stromal stem cells that produce osteoblasts and make bone. This is in keeping with reduced bone formation in older animals. Thus, we hypothesized that ENPP2 is, at least in part, an early mediator of bone formation and thus may reflect reduced bone formation with age. Since ENPP2 has not previously been shown to have a role in osteoblast differentiation, we studied its effect on bone differentiation from stromal stem cells, verified by flow cytometry for stem cell antigens. In these remarkably uniform osteoblast precursors, we did transfection with ENPP2 DsiRNA, scrambled DsiRNA, or no transfection to make cells with normal or greatly reduced ENPP2 and analyzed osteoblast differentiation and mineralization. Osteoblast differentiation down-regulation was shown by alizarin red binding, silver staining, and alkaline phosphatase activity. Differences were confirmed by real-time PCR for alkaline phosphatase (ALPL), osteocalcin (BGLAP), and ENPP2 and by Western Blot for Enpp2. These were decreased, ∼50%, in osteoblasts transfected with ENPP2 DsiRNA compared with cells transfected with a scrambled DsiRNA or not transfected (control) cells. This finding is the first evidence for the role of ENPP2 in osteoblast differentiation and mineralization.

**NEW & NOTEWORTHY** We report the discovery that the ecto-nucleotide pyrophosphatase/phosphodiesterase, ENPP2, is an important regulator of early differentiation of bone-forming osteoblasts.

## Graphical abstract

**Figure:**
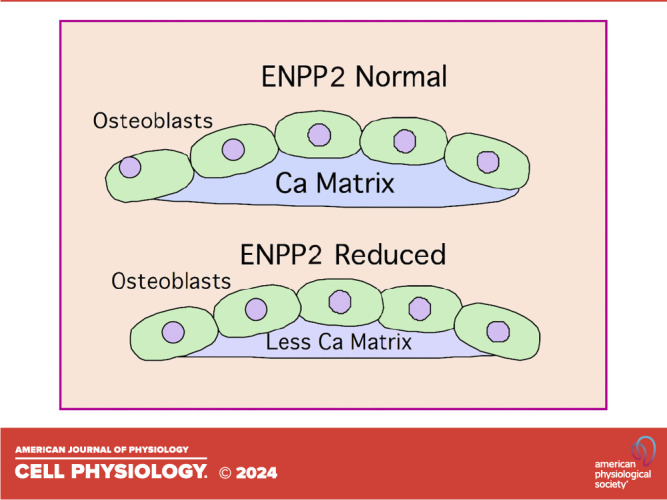

## INTRODUCTION

Bone components are synthesized by transport across an epithelial-like layer of osteoblasts connected by tight and gap junctions (1–4). Osteoblasts produce and secrete the major bone structural proteins, type I collagen, and osteocalcin (BGLAP). The extracellular collagen trimers and osteocalcin occur in layers at alternating right angles forming an extracellular matrix onto which mineral is added (4). This dense collagen matrix is mineralized as the osteoblasts provide calcium, phosphate and remove protons (2–4). We showed recently that stromal stem cells from bone (also called mesenchymal stem cells) reproduce the epithelial-like layer when grown on perforated polyethylene terephthalate (PET) membranes and very efficient differentiation of essentially pure osteoblasts in Dulbecco’s modified Eagle’s medium at pH ∼8, including high trans-epithelial resistance and trans-epithelial pH gradient (5). In this paper, we used stromal stem cells differentiated on PET membranes to study how a novel phosphate-degrading enzyme affects bone differentiation.

The most abundant enzyme for mineral production has long been known to be the liver-kidney-bone alkaline phosphatase (ALP) (6), which is also useful as a marker for osteoblast differentiation (7). It catalyzes phosphate ester hydrolysis reactions, producing phosphate that is incorporated into bone. Other enzymes that regulate phosphate production occur in osteoblasts. These include the ankylosis homology protein (ANKH or ANK) (8), which also regulates bone formation with variable opinions about the mechanism which may be ATP efflux (9) and may regulate important adipocyte-osteoblast fate decision (10). Another potentially important family of phosphatases are the ecto-nucleotide pyrophosphatase/phosphodiesterases (ENPPs) (11–12), which are weakly homologous aside from a conserved phosphodiesterase domain. ENPP1 is a transmembrane protein; ENPP2 is secreted and is alternatively called Autotaxin. ENPP1 is known to regulate bone mass in vivo and in vitro (12). It also regulates the calcification of nonbone tissues including arterial walls (13) and modulates bone formation in vitro in MC3T3E1(C4) osteoblast-like cells (14). ENPP2 has been reported to occur in osteo/chondrogenic development (15) but the role of ENPP2 in the regulation of bone formation was unknown. Gene-screens comparing osteoblasts from young and old mice suggested a possible role of ENPP2 in bone differentiation. Using siRNA to knock-down the expression of ENPP2 confirmed its direct role in bone differentiation.

## MATERIALS AND METHODS

### Animals

C57BL/6J mice were from The Jackson Laboratory. Young animals were 3–4 mo old; old animals were 17–18 mo old. Equal numbers of male and female animals were used. No sex-specific differences were noted. The animal protocol was approved by the University of Pittsburgh Institutional Animal Care and Use Committee. All animal experiments comply with the National Institutes of Health guide for the care and use of Laboratory animals.

### Reagents and Cells

Media, reagents, and chemicals were from Thermo Fisher (Waltham, MA) or as stated. Bone marrow-derived stromal stem cells (SSCs) were isolated from mouse femur and tibia as described (16). Briefly, cells were flushed using a 10-mL syringe with RPMI medium with 10% FBS. Erythrocytes were removed with cell lysis buffer. Cells were plated overnight to remove fibroblasts. Nonadherent cells were re-plated in RPMI for 72 h, then the medium was replaced with Mesenchymal Stromal Cell Culture expansion media (Mesencult, Stem Cell Technologies, Vancouver, BC, Canada). After SSCs grew well, Mesencult medium was changed to DMEM with 1.0 g/L glucose and 10% FBS (proliferation medium). For SSCs differentiation into osteoblasts, cells at 90% confluence, were placed in osteogenic differentiation medium (proliferation medium with 30 μg/mL l-ascorbic acid, 10 mM 2-phosphoglycerol and total calcium concentration 2.0 mM). SSCs were differentiated in osteogenic differentiation medium for 2 or 3 wk on polyethylene terephthalate (PET) membrane inserts with 0.4-μm perforations that optimize mineral transport into the matrix and acid transport (5).

### Flow Cytometry

Characterization of SSCs by flow cytometry using a FACScalibur instrument (Becton Dickinson, San Diego, CA), as described (5). Mouse anti-human monoclonal antibodies against CD44, Sca-1, and CD45, all conjugated with fluorescein isothiocyanate (FITC), were applied to the SSCs in proliferation. Isotype-matched mouse antibodies conjugated with FITC were used as negative controls to determine background staining. Data analysis used Cell Quest Software (Becton Dickinson).

### Alkaline Phosphatase Activity, Collagen, and Alizarin Red or von Kossa Mineral Staining

Alkaline phosphatase activity was determined using 7-bromo-3-hydroxy-2-naphthoic-O-aniside (naphthol AS-BI phosphate) substrate, reacted with fast blue to precipitate blue insoluble product, at pH 9.5 (leukocytes alkaline phosphatase kit, Sigma). Matrix mineralization was evaluated using 2% alizarin red S (alizarin red) (3,4-dihydroxy-9,10-dioxo-2-anthracenesulfonic acid) that binds to calcium to form a complex that appears in red nodules under the microscope, or alternatively using 2% AgNO_3_ (von Kossa solution); the silver is deposited in calcified matrix and reduced by UV light as metallic silver. Sirius red and fast green staining were used to distinguish collagen from noncollagenous proteins (Sirius red/fast green collagen staining kit, (Chondrex, Woodinville, WA). Sirius red specifically binds the helical structure of collagen giving a pink staining while other material is stained in green. Digital images were acquired at 600 dpi using a Umax Power-Look III Flatbed Scanner.

### Gene Array

SSCs from four young and four old mice were differentiated in osteogenic medium for 2 wk. Genome-wide expression analysis used the Clariom S Array Mouse (Thermo Fisher). Differentially expressed genes were defined by absolute fold change higher than 1.2 and *P* < 0.05. *P* values were further adjusted by Benjamini–Hochberg method to control the false discovery rate (FDR).

### RNA Isolation and Real-Time PCR

Total RNA, isolated by oligo dT affinity (RNeasy, Qiagen, Hilden Germany), was reverse transcribed using random hexamers and reverse transcriptase (SuperScript III, Invitrogen, Thermo Fisher). Real-time PCR used an MX3000P instrument (Stratagene, San Diego, CA) with SYBR green (Thermo Fisher) to monitor DNA synthesis. Reactions were run in duplicate, in 25 µl reaction volume with Brilliant III Ultra-Fast SYBR Green QPCT Master Mix (Thermo Fisher), 250 nM primers, and cDNA. After 10 min at 95°C, the mixture was amplified in cycles of 30 s at 95°C, 30 s at 59°C, and 1 min at 72°C. The size and specificity of products were verified by agarose gel electrophoresis. Product abundance relative to controls was calculated assuming linearity to log (initial copies). Primers are shown in Table 1.

**Table 1.** Mouse PCR primers

ALP (alkaline phosphatase) – Product 131 bp
F – 5′- ATCGGAACAACCTGACTGACCCTT-3′
R – 5′- ACCCTCATGATGTCCGTGGTCAAT-3′
BGLAP (osteocalcin) – Product 118 bp
F – 5′- ACCATCTTTCTGCTCACTCTGCTG-3′
R – 5′- TATTGCCCTCCTGCTTGGACATGA-3′
ENPP2 (ecto-nucleotide pyrophosphatase/phosphodiesterase 2) – Product 71 bp
F – 5′- TGGCTTACGTGACATTGAGG-3′
R – 5′- AGTGGGTAGGGACAGGAATAG-3′
GAPDH (glyceraldehyde-3-phosphate dehydrogenase) – Product 184 bp
F – 5′- GTTGTCTCCTGCGACTTCA-3′
R – 5′- GGTGGTCCAGGGTTTCTTA-3′

### ENPP2 Knockdown by siRNA in Differentiating Osteoblasts

Expression of ENPP2 was reduced by transfecting mouse SSCs differentiating into osteoblasts with siRNA (Integrated DNA Technologies, San Diego, CA) using lipofectamine RNAiMAX and Opti-MEM (17). ENPP2 DsiRNA (40 pmol), or equal negative control DsiRNA, were transfected into 90% confluent cells. Negative control was scrambled DsiRNA. The dicer substrate siRNA was of three ENPP2-specific duplex constructs targeting three different loci on ENPP2, shown in Table 2. Transfection efficiency was confirmed using a dye labeled DsRNA; images were acquired using a fluorescent microscope Olympus IX51 with Spot software. Validation of the knockdown was by real-time PCR at 24 h, 48 h, and at 2 wk of differentiation. To ensure that ENPP2 was reduced throughout the experiment, cells were transfected on *day 1* and again on *day 7*.

**Table 2.** ENPP2 RNAi duplexes

Duplex Name	Duplex Sequences
mm.Ri.ENPP2.13.2 (Exons 13,14)	5′- GGAUGAUAUUACUUUAGTACCUGGA-3′
	3′- CACCUACUAUAAUGAAAUCAUGGACCU-5′
mm.Ri.ENPP2.13.3 (Exons 18,19)	5′- GAAGUUUGAAUCACCUGCUACGCAC-3′
	3′- ACCUUCAAACUUAGUGGACGAUGCGUG-5′
mm.Ri.ENPP2.13.6 (Exons 22, 23)	5′- AUGCAUUCCUUGUAACCAACAUGGT-3′
	3′- ACUACGUAAGGAACAUUGGUUGUACCA-5′

### Western Blots

Cells were lysed on ice in RIPA extraction buffer (Thermo Fisher) with proteinase and phosphatase inhibitors. Lysates were sonicated and cleared by centrifugation. Proteins were denatured in Laemmli sample buffer by boiling, separated by SDS-polyacrylamide gel electrophoresis, and were transferred to polyvinylidene difluoride membranes (Bio-Rad, Hercules, CA) by semi-dry transfer (Bio-Rad). Membranes were blocked in 5% blocking reagent (Bio-Rad) for 2 h and washed. Primary antibody was added overnight at 4°C. Enpp2 (Autotaxin) polyclonal rabbit Ab (Proteintech, 1:1,000) or β actin monoclonal mouse Ab (1:10,000, Sigma) were used. Unbound antibody was removed by washing. Labeled proteins were detected with peroxidase-linked secondary antibody (1:10,000, Jackson ImmunoResearch) for 1 h at room temperature. Blots were stripped and re-probed. Bound antibodies were visualized by enhanced chemiluminescence (Super Signal West Femto Maximum, Thermo Fisher) and detected using a gel doc system (ChemiDoc, Thermo Fisher). Protein intensity was quantified using ImageJ software (NIH, USA; Public Domain).

### Statistics

Statistical significances of data were evaluated with the Student’s *t* test with significance called at *P* < 0.05. Data are reported as means ± SD.

## RESULTS

### Genome-Wide Expression Analysis of Osteoblasts Differentiated in Vitro Showed Major Decrease in Ecto-Nucleotide Pyrophosphatase/Phosphodiesterase 2

**Figure 1.**
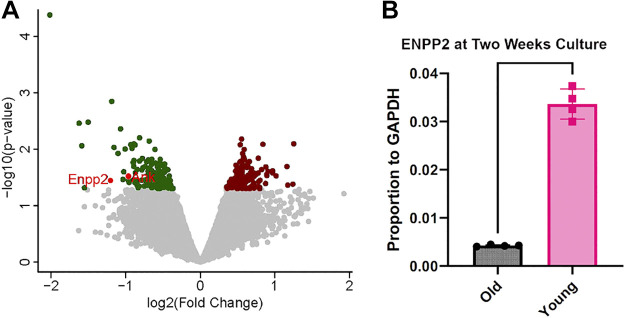
Volcano plot of differentially expressed genes with age from quadruplicate osteoblast cultures from young animals vs. quadruplicate osteoblast cultures from old animals. *A*: genes downregulated with age are shown on the left side. Those with expression such that *P* < 0.05 and fold change was >1.2 were identified. Only two phosphatase-active genes were identified, which are labeled, Ecto-nucleotide pyrophosphatase/phosphodiesterase 2 (ENPP2) and ANK (also called ANKH). ANK was previously known to affect bone formation and was not studied further. *B*: ecto-nucleotide pyrophosphatase/phosphodiesterase 2. We confirmed the effect of age by real-time PCR, *P* < 0.001.

Our interest was in phosphatase-related enzymes. Stringent criteria were applied to determine the effects of differentially expressed genes where only those with level of expression such that *P* < 0.05 and fold change was >1.2 were pursued further as shown in volcano plot (Fig. 1*A*). Only two phosphate processing enzymes, ENPP2 and ANK (also called ANKH) met these criteria and were significantly down-regulated in old mice. The result with ENPP2 was confirmed using real-time PCR from osteoblasts of young and old mice harvested from the PET membrane inserts (Fig. 1*B*). Since ANK was previously shown to regulate bone mineral (9–10), ANK was not further studied.

### Validation of Stromal Stem Cells Differentiated into Osteoblasts on PET Membrane Inserts

**Figure 2.**
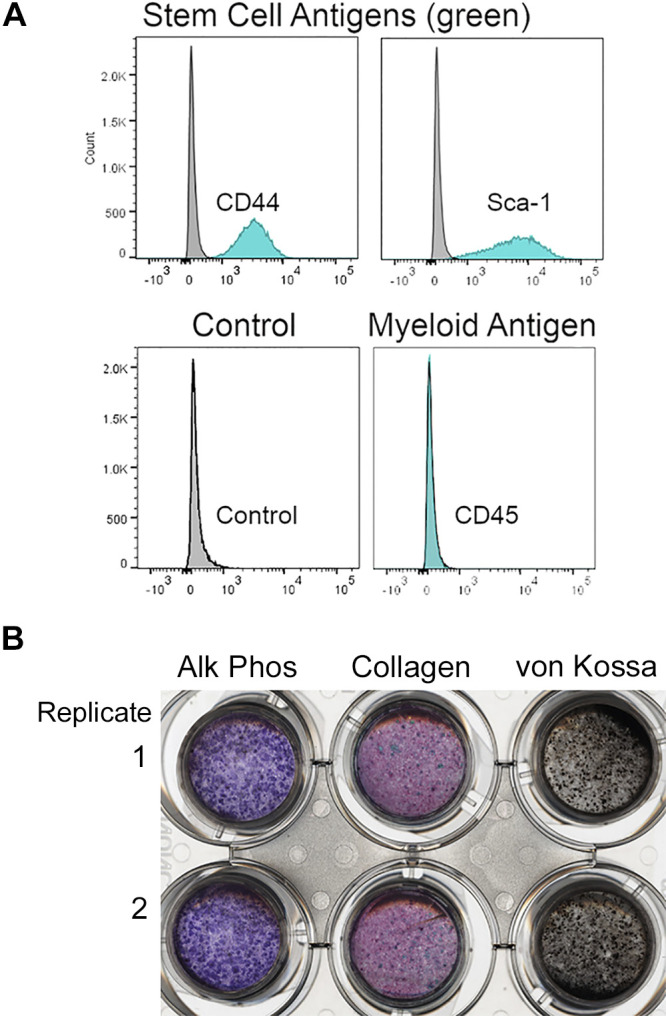
Properties of isolated stromal stem cells used. *A*: stromal stem cells were isolated from femoral bone using selective medium. With isotype controls in grey, the stem cell antigens CD44 and Sca-1 are expressed; the negative control myeloid antigen CD45 (*right lower panel*) was not expressed. CD45 is shown with the isotype control as a separate graph because the two are essentially identical (*left lower panel*). This is similar to results in the previous report (5). This is the preparation used for differentiation controls, below. *B*: differentiation of osteoblasts as stem cells when differentiated on PET membranes (5), highly uniform dense layers of osteoblasts with mineralized matrix are seen at 3 wk. This is the preparation of stem cells used for studies of gene knockdown. Shown are alkaline phosphatase activity (*left*), Sirius red/fast green collagen (*middle*) and von Kossa stains (*right*). Shorter-term differentiation is used (2 wk) subsequently to capture early effects on cell differentiation.

Cells expressed CD44 and Sca1 and were negative for CD45 (Fig. 2*A*). Osteoblasts differentiated from SSCs on PET membranes for 3 wk were analyzed by alkaline phosphatase for osteoblasts, collagen production, and von Kossa stain for mineral (Fig. 2*B*). The flow cytometry and the ability of the cells to have a high alkaline phosphatase activity with a strong mineralized collagenous matrix deposition confirmed the characterization of stromal stem cells as well as their ability to differentiate into osteoblasts.

### Transfection Efficiency of siRNA Treatment

**Figure 3.**
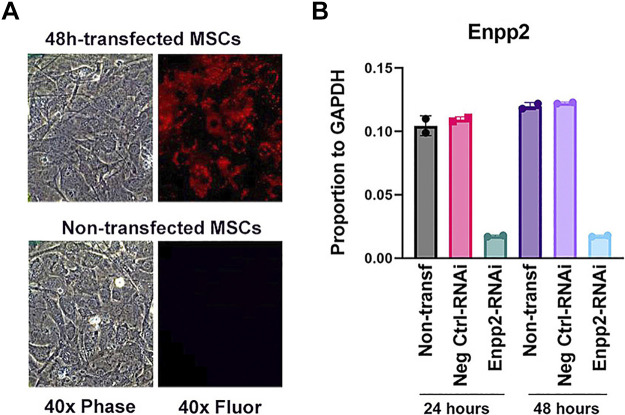
Transfection efficiency shown by fluorescence analysis. Shown are cells on PET membranes with the transfection started at the same time as differentiation. *A*: transfection control was 70–80% at 24 and 48 h of transfection. Images taken with ×40 objective are 350 × 200 µm. Images on the *left* are phase contrast, those on the *right* are red fluorescence for the siRNA label. *B*: real-time PCR validation of ENPP2 transfection. ENPP2 was reduced by 82% at 24 h and 85% at 48 h, *P* < 0.001, *n* = 2. Controls, not transfected or negative control, were not changed significantly.

Transfection efficiency measured by fluorescent transfection control was 70–80% at 24 and 48 h of transfection (Fig. 3*A*). Real-time PCR validation of ENPP2 transfection confirmed 82% ENPP2 silencing at 24 h and 85% at 48 h (Fig. 3*B*), *P* < 0.01.

### ENPP2 Plays an Important Role in Early Osteoblast Differentiation

**Figure 4.**
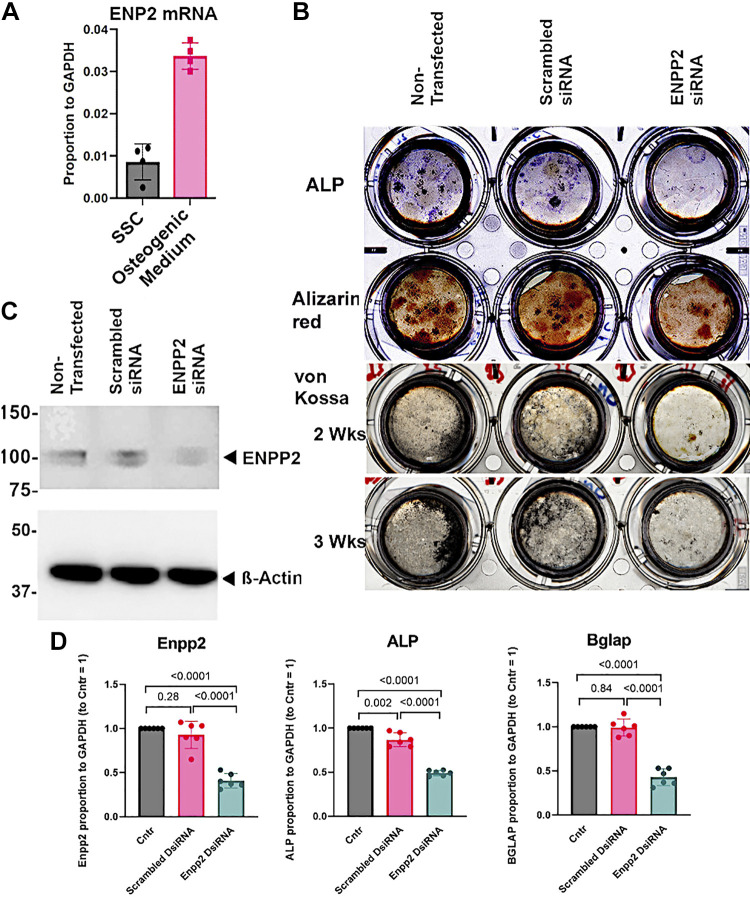
The Role of ENPP2 in osteoblast differentiation. *A*: during osteoblast differentiation, ENPP2 mRNA increases ∼ fourfold (*P* < 0.001). *B*: silencing of ENPP2 with DsiRNA decreased osteoblast differentiation on PET membranes. With transfection on the second day after plating stromal stem cells, silencing ENPP2 decreased alkaline phosphatase activity, alizarin red staining, at 2 wk of differentiation, and mineralization (von Kossa) after 2 and maintained in 3 wk of additional cell culture. Un-transfected and nonsense transfected controls and knockdown cells on PET membranes are shown, *left* to *right*, respectively. *C*: Western analysis of ENPP2 in nontransfected cells and cells with ENPP2 siRNA. Results at 2 wk of differentiation are shown, illustrating ∼60% decline in ENPP2 protein. This was verified by PCR analysis in part (*D*), below. *D*: real-time PCR of cells harvested from PET membranes after 2 wk of differentiation. Nontransfected and nonsense transfected cells have minor differences; ENPP2 mRNA was downregulated 60%, alkaline phosphatase 53%, and osteocalcin (BGLAP) 40%. Two separate experiments with duplicates (*P* < 0.001 siRNA vs. scrambled control or nontransfected).

Expression of ENPP2 is higher in osteoblasts compared with SSCs, confirming a role for ENPP2 in osteoblast differentiation (Fig. 4*A*). Silencing of ENPP2 with DsiRNA at day 2 decreased osteoblast differentiation, evaluated by ALP activity, alizarin red and von Kossa staining (Fig. 4*B*). The ENPP2 knockdown was confirmed by Western analysis; one of two blots with similar results is shown (Fig. 4*C*). Differences in differentiation at 2 and 3 wk in ENPP2 siRNA were confirmed by real-time PCR (Fig. 4*D*). After 2 wk of osteogenic differentiation, ENPP2 expression was down-regulated 60%. ALP activity was decreased by ∼half, bone gla protein (BGLAP) was reduced by 40%. Differences were consistent, all *P* < 0.001, in three experiments performed each in duplicate. Osteoblasts transfected with a scrambled DsiRNA were not significantly changed relative to control cells. As additional controls, cells were kept for 3 wk with ENPPsiRNA in differentiation medium; development remained suppressed (Fig. 4*B*, *bottom row*). In a further control, mature osteoblasts expressing high levels of alkaline phosphatase at 3 wk in differentiation medium (Fig. 2*B*) were transfected with ENPP2 DsiRNA; no significant effects on ALP, bone gla protein, or mineralization were seen (not illustrated).

## DISCUSSION

This is the first demonstration of the role of ENPP2 in osteoblast differentiation. Our genome-wide screening analysis showed two phosphatase enzymes that varied with age in mice, age being a strong cause of bone loss (Fig. 1). One gene identified, ANK, is well known to be related to bone formation and was not further studied; the other gene, ENPP2, had not been implicated directly in bone differentiation. In earlier work, we reported that ENPP2 was downregulated with age (18), but its function was unclear.

Briefly, ENPP2 was not identified in earlier studies of mineral transport and bone formation (4). ENPP2 expression is reported in muscle, osteo/chondrogenic, and tooth development (15, 19). On the other hand, the expression of ENPP2 was shown in comprehensive analysis of ENPPs to be down-regulated in PCR assays of osteoblasts differentiated in acidic conditions (19), with its expression related to Wnt/β-catenin signaling (20). In this regard, acid transport into osteoblasts and phosphate concentration are key requirements for bone mineral synthesis (4).

Our results here strongly implicate ENPP2 as playing an important role in early osteoblast differentiation. This was confirmed using real-time PCR of expression (Fig. 1*B*) and by siRNA knockdown of ENPP2 in cells grown on PET membranes (Fig. 4), a sensitive and highly reproducible method reported recently (5). The effect of the ENPP2 knockdown was durable for 3 wk (Fig. 4*B*, *bottom row*).

Advantages of the methods used include uniformity of starting cells (Fig. 2). Effects on early differentiation we used differentiation times of 2 to 3 wk, with ENPP2 siRNA treatment beginning within 2 days of plating stromal stem cells (Fig. 4*B*). These experiments were done in three different cell cultures in duplicate and were very reproducible. Confirmation that the mechanism relates to early differentiation was obtained by repeating ENPP2 siRNA exposure in fully differentiated osteoblasts (at 3 wk of differentiation before treatment, as shown in Fig. 2*B*). In these mature osteoblasts no effect on osteoblastic features was seen with transfection of ENPP2 siRNA (not illustrated).

The family of ENPPs contains at least seven elements, which are quite variable except for a phosphodiesterase domain that has ∼ 40–50% identity between genes. ENPP1,3,4, and 5 specialize on nucleotide-base substrates (e.g., diadenosine triphosphate and cyclic nucleotides) while ENPP2,6, and 7 prefer lysophospholipids. A common part is the action toward a phosphate diester bond, which is the defining feature of the family (12). ENPP1 was shown to mediate calcification in non-bony sites, including aorta (13). Humans with heterozygous loss of function pathogenic variants in ENPP1 develop early-onset osteoporosis (21, 22). That finding was confirmed in murine models of ENPP1 deficiency reported by multiple investigators (21, 23–26). Moreover, recent studies of novel murine models of ENPP1 deficiency demonstrate that ENPP1 regulates bone mass via a catalysis-independent mechanism (27). Although ENPP1 effects on bone formation were reported in an osteoblast-like cell line (14), it was not identified in our genome-wide analysis for the osteoblasts differentiated from stromal stem cells of young versus old mice.

ENPP2 was not studied in bone differentiation prior to this report, although its occurrence in cartilage and bone was known (15). Although the identification of the extracellular phosphatase ENPP2 is a valuable contribution to the mechanism of mineral formation, the authors believe that is likely that there are additional genes, not yet identified, in bone formation, in that a mechanism for the massive secretion of phosphate through osteoblasts (4) remains unclear.

We conclude that ecto-nucleotide pyrophosphatase/phosphodiesterase 2 is an important stimulus of early osteoblast differentiation for bone formation and mineral accumulation by alkaline phosphatase and bone mineral formation labeling (Fig. 4*B*). PCR for alkaline phosphatase and the matrix component bone gla protein (osteocalcin) confirmed the cell differentiation results.

## GRANTS

This work was funded in part by BX002490-06A1 from the Department of Veteran’s Affairs, USA, and by R01 AR076146-01 from the National Institutes of Health, USA.

## DISCLOSURES

No conflicts of interest, financial or otherwise, are declared by the authors.

## AUTHOR CONTRIBUTIONS

I.L.T., Q.C.L., J.L., P.H.S., and H.C.B. conceived and designed research; I.L.T., Q.C.L., and H.C.B. performed experiments; I.L.T., Q.C.L., S.L., P.H.S., and H.C.B. analyzed data; I.L.T., Q.C.L., S.L., J.L., and H.C.B. interpreted results of experiments; I.L.T., S.L., and H.C.B. prepared figures; I.L.T. and H.C.B. drafted manuscript; I.L.T., P.H.S., and H.C.B. edited and revised manuscript; H.C.B. approved final version of manuscript.
